# Gastroduodeno-plasty performed by distal gastric transection.- A new technique for large duodenal defect closure

**DOI:** 10.1186/1750-1164-6-6

**Published:** 2012-08-08

**Authors:** Martin Büsing, Hassan Shaheen, Raute Riege, Markus Utech

**Affiliations:** 1 Department of General and Visceral Surgery, Klinikum-Vest, Knappschaftskrankenhaus, Recklinghausen, Germany

**Keywords:** Duodenal defect, Bouveret’s syndrome, Gastroduodeno-plasty

## Abstract

**Introduction:**

Duodenal ulcer lesions can represent a surgical challenge, especially if the duodenal wall is chronically inflamed, the defect exceeds a diameter of 3 cm and the ulceration is located in the second part of the duodenum.

**Patient and method:**

We present the case of a 70-year-old male, who suffered from a 3 x 4 cm duodenal defect caused by duodenal pressure necrosis due to a 12.5 x 5.5 x 5 cm gallstone. Additionally, this stone caused intestinal obstruction (Bouveret’s syndrome) and bleeding with signs of shock. Besides the gallstone extraction, the common bile duct was drained by a T-tube and the duodenal defect closure was performed by a gastroduodeno-plasty and Bilroth II gastroenterostomy. The postoperative phase was uneventful. The reconstructed duodenum was endoscopically accessible and showed no pathological findings on follow-up.

**Conclusion:**

The reconstruction of a large defect (> 3 cm) of the second part of the duodenum is safely feasible by a gastroduodeno-plasty. The critical gastroduodenal anastomosis can be protected by duodenal decompression, achieved by placing a T-tube in the common bile duct.

## Introduction

While the vast majority of duodenal ulcerative lesions with a defect range from 1 to 2 cm in diameter can be relatively securely sealed by a primary surgical closure, large tissue defects (> 3 cm), especially in the second part of the duodenum, represent a special challenge in visceral surgery. If large duodenal defects are combined with a chronic mural inflammation, mobilization of the duodenum by the Kocher maneuver, to achieve a tension-free suture line, becomes almost impossible. For this rare but critical situation, surgical practice techniques as using Roux-en-Y reconstruction, omental patch-plasty, jejunal serosal patch, and stomach transection by using a stapler or furthermore draining the duodenum and stomach by using catheters, combined with a possible bile drainage are recommended [1]. Here we describe a novel approach employing the technique of gastroduodeno-plasty using the transected distal portion of the stomach to close a large defect of the anterior duodenal wall in the second portion of the duodenum following gallstone erosion and penetration leading to gastric outlet obstruction (Bouveret’s syndrome) and hemorrhagic shock.

## Case report

**Figure 1:**
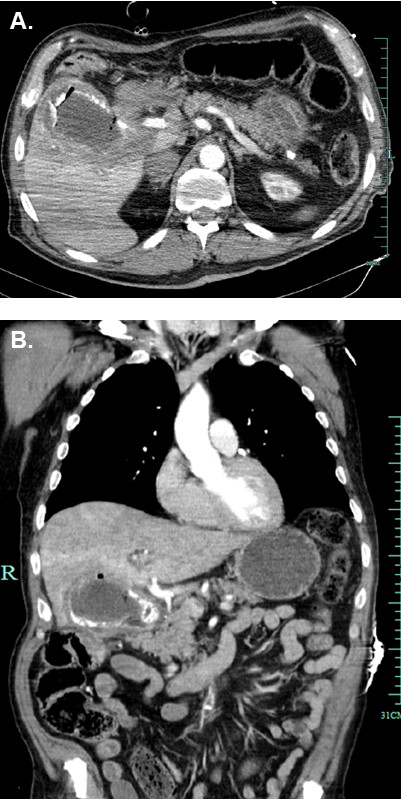
**Initially performed trauma computer tomography body scan.** (**A**) Axial intravenous contrast-enhanced CT scan and (**B**) coronal reformatted image of the anterior abdomen demonstrate a huge gallstone and an upper-abdominal process, which raised the suspicion of a perforated gallbladder.

We report the case of a 70-year-old male patient who was admitted through the emergency department with the diagnosis of syncope after having collapsed at home. The past medical history included type II diabetes, coronary heart disease with a history of PTCA, and prior myocardial infarction as well as anemia of unknown etiology diagnosed 9 months ago earlier. In addition, the patient suffered from a feeling of postprandial fullness during several days prior to presentation. Blood work showed significant anemia (hemoglobin: 5.5 g/dl; normal values: 10 to 13 g/dl). The computer tomography revealed a bilateral mandibular fracture (following the fall at home) and an upper-abdominal mass, which raised the suspicion of a perforated gallbladder (Figure 1).

The first step in treating this patient was stabilizing his vitals in the intensive care unit. Multiple blood units were administered. Upper gastrointestinal endoscopy revealed a pyloric stenosis and a postpyloric duodenal ulcer without evidence of active bleeding.

**Figure 2:**
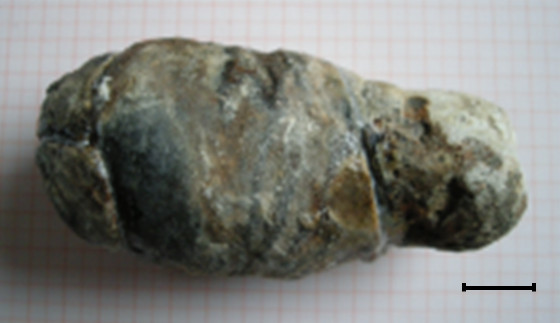
**Reassembled gallstone.** The desiccated and reassembled gallstone had an initial size of 12.5 x 5.5 x 5 cm; scale bar 2 cm.

**Figure 3:**
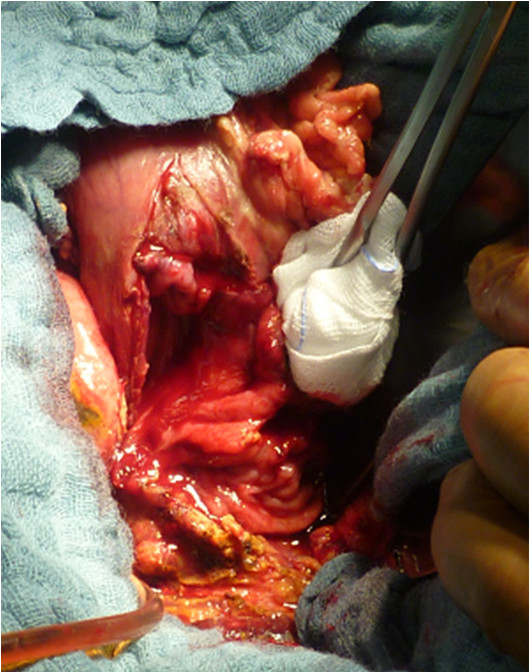
**Intraoperative situation of the duodenal penetration caused by the enormous gallstone.** The size of this defect was about 3 x 4 cm. Due to this and to the poor tissue conditions, mobilization of the duodenum and direct closure of the defect was impossible.

**Figure 4:**
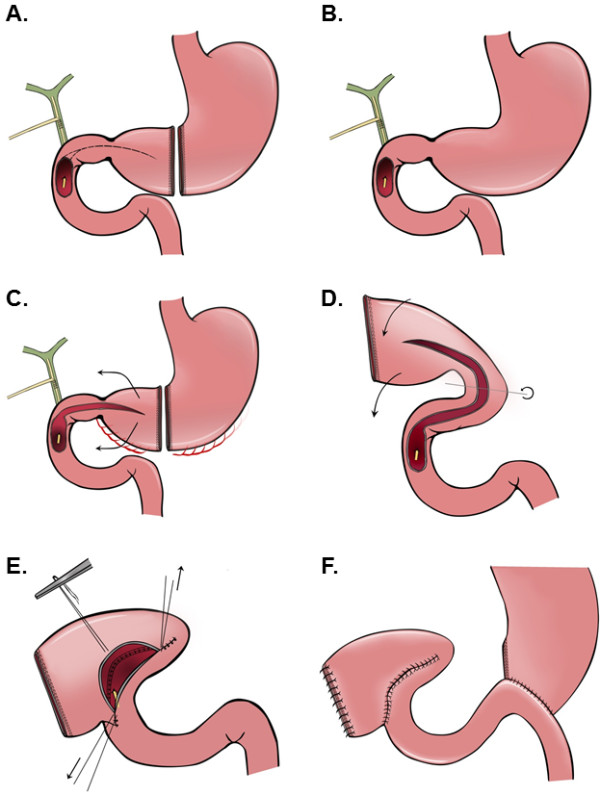
**A schematic illustration of performing a gastroduodeno-plasty to cover a large duodenal defect by the distal part of a transected stomach.** First a T-tube is placed into the common bile duct to decompress the duodenum (**A**). To mobilize the pyloric antrum the stomach is transected at the level of the Angular incisure by using a linear stapler (**B**.) The lesser curvature of the stomach is completely mobilized. And the greater curvature is mobilized while preserving the right gastroepiploic vessels. Beginning from the cranial edge of the duodenal defect the front wall of the proximal duodenum is widely opened dividing the pyloric canal and antrum (**C**). The distal transected stomach and duodenal defect are approximated by a 180 degree rotation of the distal stomach (**D**). The back wall of the anastomosis is sewn by single sutures. Corner stitches are placed and the front wall is completed using interrupted sutures (**E**). The Stapler line of the distal transected stomach is reinforced by seroserosal oversewing . The reconstruction of the gastrointestinal tract is performed in a retrocolic fashion according to Billroth II by using the first jejunal loop so that the afferent loop remains short (**F**).

After a stabilizing the patient he underwent explorative laparotomy. Intraoperatively there was a suspicious process in the right upper abdomen. The greater omentum and the hepatic flexure of the colon were adhesed to the liver. Gradual mobilization was required to identify the gallbladder. A significantly enlarged gallbladder was noted. The tissues in this area were extremely hard and fibrosed. Similar hostile conditions were found in the region of the duodenum. At this point the etiology of this process was unclear. The gallbladder wall was opened with the diathermy and a monstrous stone surrounded by a narrow purulent hem was visualized. In order to get access to this gallstone, dissection with removal of portions of the gallbladder wall was required. Finally using a spoon the outrageous, fragmented gallstone was removed. The weight of the stone was 180 grams; the size of the reassembled stone was 12.5 x 5.5 x 5 cm (Figure 2). After extracting the gallstone a significant defect of the anterior duodenal wall was identified (Figure 3). The size of this defect was about 3 x 4 cm. Due to this and to the desolate tissue condition, mobilization of the duodenum with direct closure of the defect was impossible. Therefore an alternate approach was required. For this firstly the cystic duct was located and a fogarty-catheter was used to identify the ampulla of Vater. Next, a transduodenal papillotomy was performed and the common bile duct was explored. The path of the common bile duct could be tracked in the inflamed and damaged hepatoduodenal ligament. Following this maneuver choledochotomy was possible and a decompressing T-tube was inserted. Choledochotomy closure was performed by using a monofilament resorb able suture 4–0 and at the end of the procedure the T-drain was exteriorized through the upper right abdominal wall (Figure 4A). To achieve defect closure of the duodenum distal gastric transection with rotation of the transected stomach over the duodenal defect was performed (Figure 4). For this purpose, the greater curvature was dissected from the pylorus towards the gastric body, and the stomach was transected at the level of the incisura using a linear stapler (Figure 4B), whilst preserving the right gastroepiploic artery and vein. The lesser curvature of the stomach was also completely mobilized. The vascular perfusion of the stomach was excellent due to the preserved gastroepiploic arcade. Starting from the cranial edge of the duodenal defect, an enterotomy of the proximal duodenum was performed dividing the pyloric canal and antrum using diathermy (Figure 4C). After placing several stay sutures the transected distal stomach was anastomosed to the duodenal defect by rotating the distal stomach and approximating the duodenal defect (Figure 4D, E). Due to this maneuver the defect was completely covered by the rotateddistal stomach. The overlapped distal stomach pexied to Gerota’s fascia by several stiches (Figure 4F) and a Jackson-Pratt drain was placed close to the anastomosis. The reconstruction of the gastrointestinal tract was performed using a retrocolic approach according to Billroth II by using the first jejunal loop so that the afferent loop was quite short.

The patient’s postoperative phase was uneventful. The T-tube decompressed the bile system draining up to one liter of bile per day. There was no evidence of a duodenal fistula throughout the patient’s hospital stay. A postoperative cholangiography showed an unremarkable bile duct system with good flow into the duodenum. The T-tube was removed on the postoperative day twelve. The patient tolerated an oral diet well at the point of discharge home.

Fullow-up gastroscopy was performed six weeks after the initial surgery. It was possible to examine the duodenum using retrograde intubation of the afferent limb of the Billroth reconstruction. The anterior duodenal wall in the region of the previous defect was clearly identified and biopsies were taken for histologic evaluation. The findings showed normal gastric and duodenal mucosa. The longstanding anemia had resolved. The serum gastrin levels were shown to be within normal limits (39 pg/ml: normal limits <108 pg/ml), while the patient was on proton pump inhibitors. After 6 months this study was repeated and again in the normal range (53 pg/ml) without using proton pumps inhibitors for four weeks.

## Discussion

This case is remarkable due to stone size and extension of the duodenal defect. To our knowledge this is the first time a duodenal defect of this size and location was covered using transposition of transected distal stomach. The dimension of the retrieved stone with a size of 12.5 x 5.5 x 5 cm and weighing 180 grams make it a rarity. The biggest stone previously documented in the literature had a size of 10 cm [2]. Bouveret ’s syndrome, describing the symptoms related to upper intestinal gallstone obstruction was first described in the 17^th^ century by Bartolin. Later Leon Bouveret published two cases in Revue de Medicine in 1896 [3]. Until 1993 about 300 cases have been reported in the literature [4].

In our case the patient suffered from postprandial fullness as a symptom of intestinal obstruction only a couple of days before the acute event. Diagnosis was made by endoscopy during the hemorrhagic shock. Gallstone related hemorrhage has been previously described in only a few cases. Of course, as part of the stone penetration bleeding from the gallbladder, the duodenal wall or from the cystic artery can arise [2 ,4-7]. The endoscopic image may lead to misinterpretation diagnosing only a penetrating duodenal peptic ulcer [8].

Usually when duodenal penetration by a gallstone occurs the stone’s size is usually only about 2.5 cm in diameter and typically results in a gallstone ileus [9]. The resulting cholecystoduodenal fistula can frequently be closed by direct suture repair. Larger tissue defects of the anterior duodenal wall (> 3 cm) are problematic, especially when mobilization of the duodenum by Kocher maneuver is impossible due to inflammatory tissue conditions [1,10,11] A number of different surgical techniques for duodenal closure have been described [1,12,13]. The classic surgical technique, Graham patch [14], was not possible in this case due to the defect's size. Jani et al. demonstrated in a prospective randomized study of 100 patients with duodenal perforation and defect size of 2 to 3 cm that using either a Graham patch or a omentum plug that the omentum plug lead to less postoperative leaks and overall decreased mortality [15]. Therefore the omentum plug is a promising treatment approach for a duodenal perforation size of 2 to 3 cm. However in this case the defect exceeded this dimension and using an omentum plug would have led to complete obstruction of the duodenum. In 2009 Lal et al. described the method of tube duodenostomy for the management of giant duodenal ulcer perforation. This group reported the outcomes of 20 patients treated by gastroduodenostomy and draining the digestive fluids by placing a gastric, a retrograde duodenal tube and inserting additionally a jejunal feeding tube and had a great outcome. The first step in this surgical approach was the Kocher maneuver of the duodenum decreasing tension at the repair site [16], this was not possible in our case, we required an adequate amount of well mobilized tissue material to cover the defect and therefore we mobilized and transected the pyloric antrum and used it as a flap instead.

This technique has so far not been mentioned in the literature and therefore we consider it unique. The principles of decompressing the duodenum and achieving a tension-free anastomosis are preserved by this surgical technique. Decompression of the duodenum is achieved by bile draining using a T-tube and by dividing the stomach. By transecting the distal stomach the passage of food is avoided and the critical anastomosis in the duodenum only has contact with pancreatic secretions. Previously described methods to achieve the same goal include gastric resection, stomach partition by stapling, gastroenterostomy, or a choledochojejunostomy or by draining the stomach and the duodenum [10,11,17]. Further approaches in treating duodenal defects are using Roux-en-Y reconstructions or a pedicled stomach flap. Until now the latter method has only been explored experimentally [12,18]. In our approach we used the pedicled distal stomach which leads to a very good result. Even in extreme inflamed conditions of the duodenal region, the stomach is usually not affected. The crucial prerequisite to perform a gastoduodeno-plasty is the integrity of the right gastroepiploic arcade. By preserving these vessels, almost any portion of the stomach can be used to cover a duodenal wall defect. The 180° rotation of the stomach did not affected the stomach perfusion at any time.

The reconstruction of the gastrointestinal tract was intentionally performed using the first jejunal loop according to *Billroth II* in modification of *von Mikulicz-Radecki*. This gastrojejunostomy allows the duodenum to be reached endoscopically using a retrograde technique. Six weeks after surgery we performed a follow-up endoscopy; the blind end of the duodenum which is covered by the distal stomach could easily be accessed. The transduodenal sphincteroplasty was widely patent and easily catherterized. In the area of the gastroduodeno-plasty no mucosal inflammation or ulceration were observed and normal histopathology was reported from the routine surveillance biopsies

Possible criticism of this technique is the alteration of the pathophysiological conditions within in the new duodenum following the gastroduodeno-plasty. Due to the direct contact of the pyloric antrum mucosa to the alkaline duodenal secretions a significant increase in gastrin stimulation would be expected leading to an increased risk of peptic lesions in the gastrointestinal anastomosis or jejunum. This hypothesis is supported by animal studies on the pathophysiology of ulcer formation conducted in the 70’s and 80’s [19]. However, in our case we treated our patient with 6 weeks of proton pump inhibitor medication; following this the medication was ceased. Serum gastrin levels taken at 6 weeks and 6 months both revealed normal levels; therefore we conclude that the expected effect is not significant.

## Conclusion

This is the first description of covering a large duodenal wall defect (> 3 cm) by performing a gastroduodeno-plasty with rotation of the transected stomach in a case where the duodenum could not be mobilized by a Kocher maneuver.

## Consent

This section should provide a statement to confirm that the patient has given their consent for the Case reports to be published. The editorial office may request copies of the informed consent documentation at any time. We recommend the following wording is used for the consent section: “Written informed consent was obtained from the patient for publication of this Case report and any accompanying images. A copy of the written consent is available for review by the Editor-in-Chief of this journal.”If the patient has died, then consent for publication must be sought from the next of kin of the patient. If the patient is a minor, or unable to provide consent, then consent must be sought from the parents or legal guardians of the patient. In these cases, the statement in the 'Consent’ section of the manuscript should be amended accordingly.

## Competing interests

The authors declare that they have no competing interests.

## Authors’ contributions

MB, HS, RR and MU received and followed the patient; MU wrote the manuscript. MB operated and HS, RR and MU participated to the operation and the follow-up. MB, HS, RR helped to draft the manuscript. All authors read and approved the final manuscript.
